# Prevalence and association between obesity and metabolic syndrome among Chinese elementary school children: a school-based survey

**DOI:** 10.1186/1471-2458-10-780

**Published:** 2010-12-22

**Authors:** WeiJia Liu, Rong Lin, AiLing Liu, Lin Du, Qing Chen

**Affiliations:** 1 Department of Epidemiology, School of Public Health and Tropical Medicine, Southern Medical University, Guangzhou 510515, China; 2 Department of School Health, Guangzhou Center for Disease Control and Prevention, 23 Zhongshan 3rd, Guangzhou 510080, China; 3 National Institute for Nutrition and Food Safety, Chinese Center for Disease Control and Prevention, 29 Nanwei Road, Beijing 100050, China

## Abstract

**Background:**

China has experienced an increase in the prevalence of childhood overweight/obesity over the last decades. The purpose of this study was to examine the prevalence of obesity and metabolic syndrome among Chinese school children and determine if there is a significant association between childhood obesity and metabolic syndrome.

**Methods:**

A cross-sectional study was conducted among 1844 children (938 males and 906 females) in six elementary schools at Guangzhou city from April to June 2009. The body mass index (BMI), waist circumference, blood pressure, Tanner stage, lipids, insulin and glucose levels were determined. Criteria analogous to ATPIII were used for diagnosis of metabolic syndrome in children.

**Results:**

Among 1844 children aged 7-14 years, 205 (11.1%) were overweight, and 133 (7.2%) were obese. The prevalence of metabolic syndrome was 6.6% overall, 33.1% in obese, 20.5% in overweight and 2.3% in normal weight children. Multiple logistic regression analysis showed that BMI (3rd quartile)(OR 3.28; 95%CI 0.35-30.56), BMI (4th quartile)(OR 17.98; 95%CI 1.75-184.34), homeostasis model assessment (HOMA-IR) (2nd quartile) (OR2.36; 95% CI 0.46-12.09), HOMA-IR (3rd quartile) (OR 2.46; 95% CI 0.48-12.66), HOMA-IR (4th quartile) (OR3.87; 95% CI 0.72-20.71) were significantly associated with metabolic syndrome.

**Conclusions:**

The current epidemic of obesity with subsequent increasing cardiovascular risk factors has constituted a threat to the health of school children in China. HOMA-IR and BMI were strong predictors of metabolic syndrome in children. Therefore, rigorous obesity prevention programs should be implemented among them.

## Background

Due to changes in childhood lifestyle characterized by the lack of physical activity and an energy dense diet, the worldwide epidemic of obesity represents a significant challenge in public health and pediatric medicine [1]. Increasing prevalence of obesity in both adults and children has been observed in many countries throughout the world [2,3]. China as well has experienced marked increases in the prevalence of childhood overweight/obesity over the last few decades. The overall prevalence of overweight and obesity among children in China were approximately 5% and 2%, respectively, in the 1980s. A total of 155 million children worldwide are overweight or obese, of which 12 million live in China in 2002 [4,5]. Many studies showed that high levels of body mass index (BMI, kg/m^2^) among children and adolescents were associated with abnormal levels of lipids, insulin, blood pressure and all components of the metabolic syndrome [6-8]. Data from the Third National Nutrition and Health Examination Survey (NHANES III, 1988-96) in the United States revealed that the prevalence of the metabolic syndrome was significantly related to weight status, being found among less than 0.1% of adolescents of normal weight (BMI < 85th percentile), but increasing to 6.8% among overweight adolescents (85th-95th BMI percentile) and being 28.7% among obese adolescents (BMI ≥ 95th percentile) [9].

The metabolic syndrome is a constellation of metabolic abnormalities characterized by a clustering of risk factors of cardiovascular disease including visceral adiposity, hypertension, hyperglycemia and dyslipidemia. Metabolic syndrome predicts premature coronary artery disease and type 2 diabetes in adult populations [10]. There is a lot of evidence showing that exposure to obesity in early life significantly contributed to the development of atherosclerosis and cardiovascular disease later in life. The most direct evidence comes from postmortem data from the Pathobiological Determinants of Atherosclerosis in Youth (PDAY) study [11-13]. However, to the authors' knowledge, the association between the metabolic syndrome and obesity has not been assessed in Chinese elementary school children.

Therefore, the purpose of this investigation was to examine the prevalence of obesity and metabolic syndrome among elementary school children in Guangzhou city and determine if there was a significant association between childhood obesity and metabolic syndrome.

## Methods

### Study design and participants

This cross-sectional study was conducted in Guangzhou city, which is one of the leading commercial cities in southern China. Data were collected from 1844 children (938 males and 906 females) 7 - 14 years of age in six elementary schools in Guangzhou from April to June 2009. We calculated the sample size based on the prevalence of overweight/obesity in children reported in other elementary school studies in other comparable cities of China. Given the fact that the prevalence of overweight/obesity was approximately 20% among children in a similar population [5], the sample size was estimated to achieve that percentage with an error lower than 0.02. The sample size consistent with this error was 1600 children.

The multi-stage stratified cluster random sampling method was applied to obtain study subjects. Step 1, three districts were randomly selected in urban areas. Step 2, in each of the 3 selected district, 2 primary schools were randomly sampled as the target schools. Step 3, in each of the 2 selected schools, 2 classes were randomly sampled as the target classes in each of the grade from grade 1 to grade 5. Step 4, all of the students in the selected classes were sampled.

The exclusion criteria included: missing BMI information; not being in the fasting state for at least 10 hours; known diabetes or other chronic diseases; the use of medication that would affect blood pressure, glucose or lipid metabolism. Of the 1878 children recruited from 6 elementary schools, 2 were not in the fasting state, 12 were missing the BMI information and 20 declined to participate. Therefore, 1844 children (938 males and 906 females), aged 7 to 14 years were examined. All subjects were examined by the investigators who were trained medical graduate students and nurses. The study was approved by the Ethical Committee of the National Institute for Nutrition and Food Safety, Chinese Center for Disease Control and Prevention. Written informed consent was obtained from parents and participating subjects.

### Anthropometric measures, blood pressure and stage of puberty

The fasting body weight, height, waist circumference and blood pressure of the school children were measured following standardized procedures. Height and weight were measured with subjects wearing light clothing and without shoes. Height was recorded to the nearest 0.1 cm with a GMCS-I type height tester (Beijing). Weight was measured to the nearest 0.1 kg using a balance-beam scale (RGT-140, weighing Apparatus Co. Ltd. Wuxi). The waist circumference was measured to the nearest 0.1 cm at the midpoint between the bottom of the rib cage and the top of the iliac crest at the end of exhalation. A MyoTape waistline measurer was employed with the subject standing without clothing covering the waist area.

Blood pressure was measured twice to the nearest 2 mmHg by a nurse using a mercury totally closed desk-top sphygmomanometer (Model XJ300/40-1, Shanghai), after the participants were seated at rest for at least 5 minutes. The first and fourth Korotkoff sounds were used to represent the systolic and diastolic blood pressure. The average of these two measurements of systolic and diastolic blood pressures was recorded.

The physical examination also included determination of the stage of puberty according to the occurrences of menarche and first nocturnal emission. All children were included to this examination.

### Determination of glucose, lipids and insulin levels

For measuring blood levels of glucose, insulin and lipids, fasting blood samples (5 ml) were obtained from subjects after approximately 10-14 hours overnight fasting. After the blood was drawn, the tubes were gently shaken and then separated by centrifugation at 3200 rpm for 10-15 min. Plasma glucose level and serum lipids were measured with a Hitachi 7600-010 Automatic Analyzer (Hitachi High Technologies Corp., Tokyo, Japan) within 4 hours after a fasting blood sample was obtained. Plasma glucose was assayed by the glucose oxidase technique. Serum total cholesterol (TC), triglycerides (TG), LDL-cholesterol (LDL-C) and HDL-cholesterol (HDL-C) were measured enzymatically. Plasma insulin level was measured by AxSYM Insulin assay.

### Determination of the presence of the metabolic syndrome

Pediatric metabolic syndrome has been reported in many populations and there are various definitions. In our study, metabolic syndrome was defined, using the criteria proposed by De Ferranti et al [14], as three or more of the following variables and cut-off points: (1) fasting TG ≥ 1.1 mmol/L (100 mg/dL); (2) HDL-C in boys < 1.2 mmol/L (45 mg/dL), in girls < 1.3 mmol/L (50 mg/dL); (3) fasting glucose ≥ 6.1 mmol/L (110 mg/dL); (4) waist circumference (WC) > 75th percentile of the same age and sex for Chinese children and adolescents aged 7-18 years [15,16]; (5) systolic blood pressure and/or diastolic blood pressure >90th percentile of the same age and sex according to the recommended blood pressure reference cut-offs for Chinese children and adolescents [17].

In 2007, the international diabetes federation (IDF) developed a simple unified definition only for children over 10 years of age [18]. So we also used the IDF definition in children 10 years and older. The IDF definition for children from 10 to 16 years included the presence of central obesity plus two of the other four factors: (1) central obese (WC ≥ 90th percentile), (2) fasting TG ≥ 150 mg/dL, (3) HDL-C < 40 mg/dL, (4) hypertension with systolic blood pressure ≥130 and/or diastolic blood pressure ≥85 mm Hg, (5) fasting glucose ≥100 mg/dL.

### Determination of insulin resistance

Previous studies demonstrated that HOMA-IR has been validated in children and adolescents to correlate strongly with insulin resistance [19]. In clinical practice or epidemiologic studies, insulin resistance is often measured by the homeostasis model assessment, not by the euglycemic insulin glucose clamp. In this study, insulin resistance was assessed by the homeostasis model assessment according to the formula: fasting insulin (μU/ml) × fasting glucose (mmol/L)/22.5 [20,21].

### Data analysis

BMI was calculated as body weight divided by the square of height (kg/m^2^). Overweight and obesity were identified by age-and sex-specific BMI cut-off points developed by the Working Group for Obesity Task Force in China (2004), overweight was defined as BMI for age-and sex-specific categories between the 85th and 95th percentile, whereas obesity was defined as BMI at the 95th percentile or higher. Normal weight children were defined as having a BMI < 85th percentile [22].

Based on the criteria proposed by De Ferranti et al. and the IDF, overweight and central obesity was defined for children as WC ≥ 75th percentile, WC ≥ 90th percentile respectively according to the age and sex-specific circumference percentile values for the Chinese children aged 7-18 years [15,16]. Hypertension was defined as a level above the 90th percentile for age and gender based on recommended blood pressure reference cut-offs for Chinese children and adolescents [17].

Data were expressed as means ± standard deviation (SD), median (interquartile range) or percentage (%). The Student's t test was used to compare statistical differences of normal variables among different groups; for skewed variables, Mann-Whitney U test or Kruskal-Wallis H test was used. A multiple logistic regression analysis was conducted to examine the relationship between metabolic syndrome as a dependent variable and Tanner, gender, HOMA-IR and BMI as independent variables. Data were analyzed using SPSS 15.0 data entry and statistical software package (SPSS Inc., Chicago, IL). A 2-tailed P value less than 0.05 was considered statistically significant.

## Results

Of the 1844 children (938 males and 906 females), 133 (7.2%) were obese, 205 (11.1%) were overweight, and 1506 (81.7%) were of normal weight. With regard to the prevalence of obesity adjusted by gender and age, there was a significant difference in the prevalence of overweight/obesity between genders and different ages. The prevalence of overweight was higher in boys (14.2%) than that in girls(8.4%) in age 10.0 - 14.9 years, but this difference did not appear in age 7.0 - 9.9-year group (11.2% for boys, 11.6% for girls).The prevalence of obesity was higher in boys (9.1%) than that in girls (6.6%) in age 7.0-9.9 years, and this difference also presented in age 10.0-14.9 years(7.0% for boys, 3.6% for girls).

Similar results were also obtained based on the IOTF definition of overweight/obesity for children [23]. That showed that the overall prevalence rates of overweight, obesity, and normal weight were 13.4%, 3.6% and 83.0%, respectively. The ratios of overweight and obesity among boys were significantly higher than that among their female counterparts (14.1% versus 12.7%; 5.3% versus 1.9%, respectively).

### Metabolic syndrome and their components

Using De Ferranti's definition, the risk factors of cardiovascular diseases such as hypertension (434/1844; 23.5%), central obesity (432/1844; 23.4%). high triglycerides (296/1844; 16.1%), and low HDL-C (292/1844; 15.8%) were frequent, while impaired fasting glucose (3/1844; 0.2%) were infrequent in this sample. None children in this survey had diabetes. Approximately 49.3% (909/1844) of the children had at least one component of metabolic syndrome and the prevalence was significantly higher in overweight/obese (92.6%) than in normal weight children (39.4%) (p < 0.01). The prevalence of two or more components of the metabolic syndrome was 21.4% (395/1844) overall, 61.2% in overweight/obese and 12.5% in normal weight (p < 0.01). Obese and overweight children had an average of 2.08 and 1.74 components of metabolic syndrome, respectively, compared with 0.55 in normal weight children (p < 0.01). The prevalence of metabolic syndrome was 6.6% (121/1844) overall, 33.1% (44/133) in obese children, 20.5% (42/205) in overweight children and 2.3% (35/1506) in normal weight children. There was not a significant difference in prevalence of the metabolic syndrome between boys and girls (Table 1). None children had the five components of metabolic syndrome. Children were divided into two groups according to the presence or absence of metabolic syndrome (Table 2). There were no significant differences in age, gender and stage of puberty. Children with metabolic syndrome had significantly higher levels of waist circumference, BMI, triglycerides, systolic blood pressure, diastolic blood pressure, Insulin, and HOMA-IR than children without metabolic syndrome, but the levels of HDL-C had the reverse results.

**Table 1:** Prevalence of the metabolic syndrome among Chinese elementary school children in different age groups and gender

Age group(years)	Boy		Girl		Total	
			
	N	%	N	%	N	%
7~	5	2.8	8	3.8	13	3.3
8~	13	5.6	21	11.2	34	8.1
9~	13	6.7	17	9.5	30	8.0
10~	22	6.6	22	6.7	44	6.6
Total	53	5.7	68	7.5	121	6.6

**Table 2:** The clinical and metabolic characteristics in children

	Without MS(n = 1723)	With MS (n = 121)
Age	9.33 ± 1.44	9.54 ± 1.26
Sex (Females, %)	48.6(838/1723)	56.2(68/121)
Pre-Pubertal(%)	97.2(1674/1723)	96.7(117/121)
Height (m)	1.35 ± 0.10	1.40 ± 0.09
WC(cm)	56.22 ± 7.43^#^	69.10 ± 8.46^#^
BMI (kg/m^2^)	16.32 ± 2.62^#^	20.55 ± 3.18^#^
SBP (mm Hg)	99.17 ± 10.87^#^	109.57 ± 12.13^#^
DBP (mm Hg)	64.78 ± 9.62^#^	72.60 ± 11.07^#^
TG (mg/dL)	59.29(44.25-79.65)^#^	124.78(104.87-164.16)^#^
HDL-C (mg/dL)	61.17 ± 11.64^#^	43.86 ± 9.32^#^
FBS (mg/dL)	82.51 ± 6.80	82.79 ± 7.68
Insulin (μU/ml)	5.70(4.05-8.10)^#^	9.85(6.48-13.85)^#^
HOMA-IR	1.17(0.81-1.67)^#^	2.13(1.26-2.84)^#^

Data are presented as means ± SD or median (interquartile range). P-values compare variables between children with and without metabolic syndrome(MS). ^#^P < 0.05.

WC = waist circumference; BMI = body mass index; SBP = systolic blood pressure; DBP = diastolic blood pressure; TG = triglycerides; HDL-C = high-density cholesterol; FBS = fasting blood sugar; HOMA = homeostasis assessment; IR = insulin resistance.

When the IDF definition was used, only 662 children were included in the analysis because the definition is restricted to children ≥ 10 years of age. The prevalence of metabolic syndrome and their components in children 10 years or older was determined using both definitions. The prevalence of hypertension, waist circumference, HDL-C and Triglycerides using the IDF definition were lower than that using De Ferranti's definition, which were 3.0% vs 22.1%, 10.1% vs 23.4%, 2.7% vs 17.8%, 4.1% vs 20.2%. However, there was a higher prevalence of fasting glucose (1.1%) using the IDF definition fasting glucose cut point than that (0.3%) using the De Ferranti's definition fasting glucose cut point(p < 0.01). Approximately 17.7% (117/662) and 52.9% (350/662) had at least one component of metabolic syndrome using the IDF and De Ferranti's definitions, respectively (p < 0.01). The prevalence of children with two or more components of the metabolic syndrome was 3.0% (20/662) and 23.0% (152/662) using the IDF and De Ferranti's definition, respectively (p < 0.01). Table 2 summarizes the metabolic syndrome rates in overweight, obese and overall using both definitions There was a lower prevalence of metabolic syndrome in overweight, obese and the whole group children using the IDF definition than using De Ferranti's definition (p < 0.01)(Table 3).

**Table 3:** Differences in identification of metabolic syndrome in children ≥ 10 years according to normal weight, overweight, obese, and overall using the IDF and De Ferranti's definitions.

Metabolic syndrome	De Ferranti's definition	IDF definition
Normal weight(n = 552)	12(2.2%)	0(0.0%)
Overweight(70)	17(24.3%)^#^	1(1.4%)^#^
Obese(n = 40)	15(37.5%)^#^	1(2.5%)^#^
Overall(n = 662)	44(6.6%)^#^	2(0.3%)^#^

P-values compare levels between children with metabolic syndrome using the IDF and De Ferranti's definitions.

# P < 0.01.

### Multiple regression analysis

Multiple logistic regression analysis revealed that BMI, and HOMA-IR increased the odds of having metabolic syndrome among children studied after adjustment for age, gender, tanner, WC, blood pressure, TG, HDL-C and fasting blood sugar (Table 4). Children had approximately 3.28-17.98 times higher odds of having metabolic syndrome with increasing quartiles of BMI and 2.36-3.89 times higher odds of having metabolic syndrome with increasing quartiles of HOMA-IR (Table 4).

**Table 4:** Multiple logistic regression analysis of the association between selected independent variables and metabolic syndrome using De Ferranti's definition.

					95.0% CI for OR	
					
	B	S.E.	**Sig**.	OR	Lower	Upper
BMI						
BMI (1st quartile)				1.000		
BMI (2nd quartile)	0.522	1.300	0.296	1.686	0.132	21.554
BMI (3rd quartile)	1.189	1.138	0.014	3.284	0.353	30.557
BMI (4th quartile)	2.889	1.188	<0.001	17.975	1.753	184.344
HOMA-IR						
HOMA-IR(1st quartile)				1.000		
HOMA-IR (2nd quartile)	0.857	0.834	0.031	2.357	0.459	12.091
HOMA-IR (3rd quartile)	0.898	0.837	0.019	2.455	0.476	12.656
HOMA-IR (4th quartile)	1.352	0.856	0.001	3.865	0.721	20.706

Dependent variable: Metabolic syndrome using De Ferranti 's definition.

1st quartile: 1%-25% percentiles, 2nd quartile: 26%-50% percentiles, 3rd quartile: 51%-75% percentiles, 4th quartile: 76%-100% percentiles.

The analysis was adjusted for age, gender, tanner, waist circumference, blood pressure, serum triglycerides, serum high-density cholesterol and fasting blood sugar.

## Discussion

The metabolic syndrome refers to a clustering of specific CVD risk factors, such as insulin resistance, abdominal obesity, impaired glucose, elevated blood pressure, elevated triglycerides, and reduced high-density lipoprotein cholesterol. Since the process of atherosclerosis begins during early childhood, the metabolic syndrome has been widely studied in pediatrics [24]. However, it is difficult to compare or contrast the prevalence of metabolic syndrome because it has been defined differently by various investigators, including Cook [9], De Ferranti [14], Goodman E [25], Weiss R [1], Cruz [26], Ford [27], and IDF [18]. In this survey, we adopted De Ferranti's definition, developed less restrictive criteria analogous to ATP III, to estimate the prevalence and distribution of metabolic syndrome among Chinese elementary school students based on its convenience in epidemiological research and clinical practice, using IDF definition as a supplement.

The results of this study suggest that there is a high prevalence of overweight/obesity in this group of Chinese school children. Compared to children with normal weight, overweight and obese children have a higher prevalence of many components of the metabolic syndrome, including central obesity, hypertension, high triglycerides, low HDL-C and high glucose. Children with overweight and obesity tend to have a cluster of multiple components of the metabolic syndrome, on average 1.75 and 2.08, respectively. Furthermore, insulin resistance and obesity are both strong predictors of the children's metabolic syndrome. These finding are consistent with previous research [28,29], suggesting that interventions to reduce obesity in school children are needed.

The rise in the prevalence of metabolic syndrome in children is one of the most alarming public health issues facing the world today. By applying the criteria developed by De Ferranti et al., the prevalence of metabolic syndrome in children is low in China compared to their USA counterparts (about 10% in the USA vs 6.6% in China), and the same as the prevalence of metabolic syndrome among overweight adolescents (about 31.2% in the USA vs 20.5% in China), possibly as a result of different genetic and lifestyle factors, such as dietary habits, physical activity patterns that require further research. Many studies showed that the prevalence of metabolic syndrome varies based on different definition, specific criteria and cutoff values for each component. This is particularly true in children. The prevalence of metabolic syndrome defined by IDF criteria was lower than that defined by De Ferranti's criteria. Only 0.3% of the children were identified with metabolic syndrome using the IDF definition while 6.6% using De Ferranti's definition in children 10 years and older. The IDF definition underestimated metabolic syndrome compared to the De Ferranti's definition in overweight, obese, normal weight and overall children. Our results are consistent with previous studies [25,30].

Recent studies suggest that obesity is associated with insulin resistance in both adults and children. Consistent with previous reports in adults and in children [31,32], we found that increased adiposity, measured by BMI was strongly correlated with increased triglycerides, insulinemia and decreased HDL-C, in both age groups and genders. Results from the multiple regression analysis showed that insulin resistance and obesity were associated with the metabolic syndrome. This might be due to several mechanisms, such as impaired insulin signaling, interference with glucose transport, decreased insulin clearance related to elevated intra-portal free fatty acids, and systemic effects of adipocyte cytokines [33]. These factors may have been operative in the development of insulin resistance in obese children. Given that changes related to increasing food portion size, consumption of high-fat, energy-dense fast foods and an increasingly sedentary lifestyle have contributed to obesity among children and adolescents, it is needed to address these changes to prevent obesity in children. Intervention may focus on school-base programs which will help to change diet or reduce sedentary behaviors.

Several limitations in this study must be noted. Firstly, the study subjects were sampled within three districts of Guangzhou city, southern China. It is appropriate to assume that the sampled population represents the elementary school students in the City of Guangzhou but is far from being a good sample at the provincial or the national level. Given the area and size of the sample, we must be very careful in generalizing the findings obtained in this study. Secondly, it was a cross-sectional analysis, and thus, the directionality of the causation cannot be established. However, appropriate analysis of cross-sectional data represents a useful initial step in identifying associations between obesity and the metabolic syndrome. Thirdly, it could be a possible source of bias as to the facts that fasting insulin and HOMA-IR was used as a surrogate marker of insulin resistance, instead of the gold standard of the hyperinsulinemic euglycemic clamp. More importantly, variables in our study are not enough to identify the relationship between obesity and metabolic syndrome, because the variability of several components of the metabolic syndrome has substantial genetic influence.

Strengths of the present study are that the estimation of metabolic syndrome is based on the specific percentile cut-points for Chinese children and adolescents instead of cut-points for US children/adolescents. There is enough evidence showing that the US criteria are not suitable for Chinese as to the higher body fat percentage at the same BMI among Asian adults. The specific definitions for Asian overweight is BMI ranging from 24.0 kg/m^2 ^to 27.9 kg/m^2 ^and obesity, BMI equal or above 28 kg/m^2 ^[34,35]. Therefore, the prevalence of metabolic syndrome in Chinese children and adolescents in our study could be more accurate. Other merits of our study including the high quality control of physical examination and the good response rate of participants.

## Conclusion

In conclusion, there is a high prevalence of overweight/obesity in the elementary school children in Guangzhou, China. A substantial number of overweight/obesity Chinese children have metabolic syndrome. The prevalence of metabolic syndrome correlates with increased BMI. In particular, this investigation found that insulin resistance and obesity are both strong predictors of pediatric metabolic syndrome. This study provides authorities and physicians with information that overweight/obese school children are at a higher risk for future metabolic and cardiovascular diseases. To address the problem of increasing prevalence of obesity and potentially deadly consequences of metabolic syndrome in Chinese children, more researches will be needed focusing on the reasons for the increase of overweight/obesity in children and interventions so as to reduce the epidemics of overweight/obesity and metabolic syndrome in the population.

## Competing interests

The authors declare that they have no competing interests.

## Authors' contributions

WJL and QC were responsible for the study design, coordinated the study conduct, data processing, statistics analysis and manuscript writing. RL coordinated the study personnel, the recruitment phase and were responsible for data processing. ALL participated in the study design and performed the statistical analysis. LD conceived of the study, and participated in its design and coordination. All authors read and approved the final manuscript.

## Pre-publication history

The pre-publication history for this paper can be accessed here:

http://www.biomedcentral.com/1471-2458/10/780/prepub
